# Differential DARC/ACKR1 expression distinguishes venular from non-venular endothelial cells in murine tissues

**DOI:** 10.1186/s12915-017-0381-7

**Published:** 2017-05-19

**Authors:** Aude Thiriot, Carolina Perdomo, Guiying Cheng, Igor Novitzky-Basso, Sara McArdle, Jamie K. Kishimoto, Olga Barreiro, Irina Mazo, Robinson Triboulet, Klaus Ley, Antal Rot, Ulrich H. von Andrian

**Affiliations:** 1 Department of Microbiology and Immunobiology & HMS Center for Immune Imaging, Harvard Medical School, 77 Avenue Louis Pasteur, Boston, MA 02115 USA; 2 The Ragon Institute of MGH, MIT and Harvard, Cambridge, MA 02139 USA; 3 Center for Immunology and Infection, Department of Biology, University of York, YO10 5DD, Heslington, York, UK; 4 Division of Inflammation Biology, La Jolla Institute for Allergy and Immunology, La Jolla, CA USA; 5 Stem Cell Program, Boston Children’s Hospital, Boston, MA USA; 6 Present address: Blood and Marrow Transplant Unit, Queen Elizabeth University Hospital, Glasgow, UK

**Keywords:** Monoclonal antibody, Microvascular endothelium, Leukocyte adhesion, DARC/ACKR1, Chemokines

## Abstract

**Background:**

Intravascular leukocyte recruitment in most vertebrate tissues is restricted to postcapillary and collecting venules, whereas capillaries and arterioles usually support little or no leukocyte adhesion. This segmental restriction is thought to be mediated by endothelial, rather than hemodynamic, differences. The underlying mechanisms are largely unknown, in part because effective tools to distinguish, isolate, and analyze venular endothelial cells (V-ECs) and non-venular endothelial cells (NV-ECs) have been unavailable. We hypothesized that the atypical chemokine receptor DARC (Duffy Antigen Receptor for Chemokines, a.k.a. ACKR1 or CD234) may distinguish V-ECs versus NV-ECs in mice.

**Methods:**

We generated a rat-anti-mouse monoclonal antibody (MAb) that specifically recognizes the erythroid and endothelial forms of native, surface-expressed DARC. Using this reagent, we characterized DARC expression and distribution in the microvasculature of murine tissues.

**Results:**

DARC was exquisitely restricted to post-capillary and small collecting venules and completely absent from arteries, arterioles, capillaries, veins, and most lymphatics in every tissue analyzed. Accordingly, intravital microscopy showed that adhesive leukocyte-endothelial interactions were restricted to DARC^+^ venules. DARC was detectable over the entire circumference of V-ECs, but was more concentrated at cell-cell junctions. Analysis of single-cell suspensions suggested that the frequency of V-ECs among the total microvascular EC pool varies considerably between different tissues.

**Conclusions:**

Immunostaining of endothelial DARC allows the identification and isolation of intact V-ECs from multiple murine tissues. This strategy may be useful to dissect the mechanisms underlying segmental microvascular specialization in healthy and diseased tissues and to characterize the role of EC subsets in tissue-homeostasis, immune surveillance, infection, inflammation, and malignancies.

**Electronic supplementary material:**

The online version of this article (doi:10.1186/s12915-017-0381-7) contains supplementary material, which is available to authorized users.

## Background

The peripheral circulatory system is segmentally organized into sparse arteries and arterioles that feed a dense capillary network drained by postcapillary venules (PCVs) that merge into collecting venules (CVs) and veins. In most tissues, each vascular segment is dedicated to a specific task – arteries and arterioles regulate blood flow, gas and nutrient exchange occurs in capillaries, and leukocyte adhesion and emigration is restricted to PCVs and CVs [1].

Although the expression of some gene products by endothelial cells (ECs) is regulated by hydrodynamic shear stress [2], there is evidence that the specialized function of venules in leukocyte adhesion is due to segmentally distinct endothelial programming, rather than hemodynamic differences [3]. Indeed, the specification of microvascular segments during embryogenesis is established before the initiation of blood flow [4]. Studies employing intravital microscopy (IVM) have demonstrated that the initial attachment and arrest of free flowing leukocytes to the microvascular wall in most tissues (except in lung, liver, and spleen) is a feature of PCVs and CVs, whereas capillaries and arterioles usually do not support leukocyte trafficking [5]. This specialization of venular endothelium is found in vertebrates ranging from agnathans to mammals [1, 6] and in both adults and fetuses [7].

Leukocyte recruitment from blood to tissues follows a well-established paradigm, the multi-step adhesion cascade, which distinguishes at least three consecutive dynamic interactions, namely leukocyte tethering and rolling, chemoattractant-induced activation, and integrin-mediated firm arrest [8]. Adherent cells then undergo intraluminal crawling followed by transmigration across the endothelial barrier [9]. These in vivo observations are consistent with reports on preferential venular expression of various adhesion molecules, the molecular nature and composition of which vary among different microvascular beds [8]. However, the underlying differentiation program that enables venular endothelial cells (V-ECs) to express leukocyte traffic molecules, but prohibits non-venular endothelial cells (NV-ECs) in capillaries and arterioles from doing so is unknown.

A handful of markers have been suggested to distinguish V-ECs from NV-ECs. One candidate is the atypical chemokine receptor DARC (Duffy Antigen Receptor for Chemokines, a.k.a. CD234, Atypical Chemokine Receptor-1 or ACKR1), which binds several pro-inflammatory CC and CXC chemokines [10, 11]. DARC regulates the activity of these chemokines by transcytosing them across the endothelial barrier [12]. DARC has been shown to delineate V-ECs in human kidney [13] and spleen [14] and high endothelial venules (HEVs) in murine lymph nodes (LNs) [15]. Recently, endothelial DARC expression was reported in experimental atherosclerosis and encephalitis on sections of the aorta [16] and in brain microvasculature [17], respectively.

Aside from ECs, DARC is also expressed in the erythroid lineage. Indeed, DARC was first described as a blood group antigen [18, 19] and its role on red blood cells (RBCs) has been studied extensively [11]. Erythroid DARC modulates the bioavailability of serum chemokines and serves as an entry receptor for *P. vivax* and *P. knowlesi*, enabling infection of RBCs by these pathogens [20, 21]. Immunostaining of human tissues has identified DARC on a few non-vascular cell types, such as Purkinje cells [22] and renal epithelial cells [23]. More recently, using a commercial polyclonal Ab against murine DARC, DARC expression was reported on bone marrow (BM) macrophages [24]. The same study reported that DARC was essentially undetectable on microvascular endothelium from a variety of mouse tissues, which appeared to be in contrast to previous observations in humans. Thus, it is currently unclear whether and to what extent DARC can serve as a reliable marker of V-ECs in mice. Indeed, presumably due to a lack of suitable reagents, a systematic analysis of microvascular DARC expression in murine tissues has not been performed so far.

Here, using a newly generated monoclonal Ab (MAb) against mouse DARC, we have explored DARC as an endothelial surface marker in a variety of murine tissues and assessed the utility of this research tool for flow cytometry, immunofluorescence microscopy, IVM, and Western blot analysis. We show that anti-DARC MAb can be used to identify and isolate ECs from PCVs and CVs in vitro and in vivo and that DARC expression is uniformly expressed and highly restricted to venules in multiple organs. Using flow cytometry, we determined that the relative abundance of V-ECs varies considerably between different tissues, possibly reflecting organ-specific differences in immune surveillance by migratory leukocytes.

## Methods

### Mice

C57BL/6 (catalog number 027) and BALB/c mice (RRID: IMSR_JAX:000651, catalog number 000651), 6–12 weeks old, were purchased from Charles River or Jackson Laboratories. DARC^–/–^ mice [25] were bred at the University of York and used as tissue donors. *Apoe* ^*−/−*^ mice [26] were obtained from Jackson Laboratories (RRID: IMSR_JAX:002052, catalog number 002052). BM chimeras were generated by irradiating C57BL/6 mice (2 × 650 Rad) followed by intravenous (IV) injection of unfractionated DARC^–/–^ BM mononuclear cells and a rest period of more than 12 weeks before use. Mice were housed under specific pathogen-free conditions in accordance with NIH guidelines. Experimental protocols were approved by the Institutional Animal Care and Use Committee at Harvard Medical School.

### Construction of expression plasmids

The entire open reading frame of murine DARC was PCR amplified from brain cDNA and subcloned into pCR4Blunt-TOPO (Invitrogen Life Technologies). A DARC-eGFP fusion construct was created by overlap extension PCR [27]. BamHI and ECORI were used to insert DARC-eGFP into pcDNA3.1 expression vector (Invitrogen). Primer sequences are provided in Table 1.

**Table 1:** Primers

Primers to create DARC-eGFP fusion protein		
DARC Fwd	5′- GCC ACC ATG GGG AAC TGT CT	
DARC Rvs	5′-GGA CTT GCC TGC AAG GGC AT	
BamH1-DARC Fwd	5′- GGA TCC GCC ACC GCC ACC ATG GGG AAC TGT CTG TAT C	
DARC-linker1 Rvs	5′- CCC TTG CTC ACC ATC TCG AGG GAC TTG CCT GCA AGG GCA TC	
DARC-linker3 Rvs	5′- CGC CGC GCT GCC GCC GCC GCC GGA CTT GCC TGC AAG GGC ATC	
GFP-linker1 Fwd	5′- TTG CAG GCA AGT CCC TCG AGA TGG TGA GCA AGG GCG AGG AG	
GFP-linker3 Fwd	5′- GGC GGC GGC AGC GCG GCG GCG ATG GTG AGC AAG GGC GAG GAG	
GFP-ECORI Rvs	5′- GAA TTC TTA CTT GTA CAG CTC GTC CAT GCC G	
Primers for RT-qPCR		
GAPDH Fwd	CCAATGTGTCCGTCGTGGATCT	
GAPDH Rvs	GTTGAAGTCGCAGGAGACAACC	
DARC Fwd	TCCGGTGGAAAACCTTTCACTA	
DARC Rvs	GCTGGTGTCAGGCTGTAGTC	
Selp Fwd	TGTTTGGCTTCTGGGATCTGGACA	From Kokkinaki et al. [69]
Selp Rvs	AGGCAGCAATTGGGTGCATACAG	
Madcam1 Fwd	GACCCATAGAAAGGAGATTCCAGTA	
Madcam1 Rvs	TGAGCCCAGTGGAGACTG	
Sele Fwd	TGAACTGAAGGGATCAAGAAGACT	From McEver et al. [70]
Sele Rvs	GCCGAGGGACATCATCACAT	
Chst4 Fwd	TGCCCCACCTCCAAACAT	From Ruddle et al. [71]
Chst4 Rvs	GACCAACGCCACGCCTGAGA	
Fut7 Fwd	GGACCTCCTCGGGCCACCTACG	From Ruddle et al. [71]
Fut7 Rvs	CGCCAAGCAAAGAAGCCACGATAA	

### Monoclonal anti-mouse DARC antibody

The rat cell line PC-12 (ATCC) was grown in F-12 K media (Gibco) with 2.5% fetal bovine serum (FBS) and 15% horse serum. The human embryonic kidney cell line HEK-293 was grown in DMEM (Corning) with 10% FBS. Both cell lines were stably transfected in 10-cm Petri dishes with 2 μg of plasmid using Lipofectamin 2000 (Invitrogen) following the manufacturer’s protocol. PC-12 cells were transfected with DARC-eGFP fusion protein and linker LG (named DARC-eGFP1) and HEK-293 were transfected with DARC-eGFP fusion protein and linker GGGGSAAA (named DARC-eGFP3). The antibiotic G418 (GIBCO) was added at a final concentration of 400 μg/mL 24 h after transfection. The selection medium was renewed every 3–4 days. Stable transfectants were further selected by cell sorting based on GFP expression. PC-12 DARC-eGFP transfectants were conditioned overnight with media supplemented with 10 mM sodium butyrate solution to boost transgene expression before immunization. Adult rats were immunized four times at 2-week intervals with 5 × 10^6^ sodium butyrate-conditioned DARC-eGFP PC-12 cells. For the first immunization, transfectants were suspended in complete Freund’s adjuvant and injected by sub-cutaneous (s.c.) and intra-peritonal (i.p.) routes; the first booster injection of transfectants suspended in incomplete Freund’s adjuvant was performed by i.p. and subsequent injections were performed without adjuvants by the i.p. route. Immunization and fusion of hybridomas were performed by Abpro Biotechnology company under a service contract. Cloning and sub-cloning of the hybridomas was performed in the Dana Farber Monoclonal Antibody Core, Boston. Immune sera were screened by flow cytometry for reactivity with DARC ectodomains using HEK-293 cells expressing DARC-eGFP fusion protein and RBCs from wildtype (WT) or DARC^–/–^ mice. Following splenocyte fusion, twelve 96-well plates were screened by flow cytometry. Two wells showed reactivity against mouse DARC. One clone producing an anti-mouse DARC MAb was isolated, expanded, and subcloned; the MAb was determined to be a rat IgG2a,k isotype. See Additional file 1: Figure S1.

### Antibodies for immunofluorescence staining

Anti-mouse DARC MAb was conjugated to Alexa Fluor dyes 488, 546, or 647. Anti-Lyve-1 (clone ALY7; eBiosciences) was conjugated to Pacific blue using a commercial kit (Molecular Probes). DAPI (Invitrogen) was used to stain nuclei in immunohistochemistry. UEA-1 lectin (Vector Labs) against mTECs was conjugated to Alexa Fluor 647. Insulin was detected using GP-anti-bovine insulin serum (Linco Research Inc.). See complete list of antibodies in Table 2.

**Table 2:** Antibodies for immunofluorescence staining

Label	Antibody	Clone	Company	Catalog number	RRID number
Alexa Fluor 488 conjugated	Anti-DARC	Clone 6B7	Generated in-house		
	anti-CD31	Clone 390 MEC13.3	Biolegend	102414 102502	AB_493408 AB_312913
	anti-PNAd	Clone MECA-79	eBioscience	53-6036	AB_10804391
	anti-MAdCAM-1	Clone MECA-367	Biolegend	120707	AB_493399
	Anti-LYVE1	Clone ALY7	eBioscience	53-0443	AB_1633415
	Rat IgG2a, κ	isotype control	Biolegend	400525	AB_389320
	Rat IgM, κ	isotype control	Biolegend	400811	AB_1659271
PE conjugated	anti-CD31	Clone 390	Biolegend	102408	AB_312903
	Anti-ICAM-1	3E2	BD Pharmingen	553253	AB_394735
	Armenian Hamster IgG	Isotype control	Biolegend	400908	AB_326594
PE-Cy7 conjugated	anti-gp38	Clone 8.1.1	Biolegend	127411	AB_10613294
	anti-TER-119	Clone TER-119	Biolegend	116222	AB_2281408
Alexa Fluor 647 conjugated	Anti-DARC	Clone 6B7	Generated in-house		
	rat IgG2a,k	isotype control	Biolegend	400526	AB_389342
APC-Cy7 conjugated	anti-TER-119	Clone TER-119	Biolegend	116223	AB_2137788
	Anti-CD45.2	Clone 104	Biolegend	109824	AB_830789
	anti-CD11b	Clone M1/70	Biolegend	101226	AB_10784810
Pacific blue conjugated	anti-CD45.2	Clone 104	Biolegend	109820	AB_492872
	Anti-CD45	Clone 30-F11	Biolegend	103126	AB_493535
	Anti-CD31	Clone 390		102422	AB_10612926
Brilliant violet 510 conjugated	Anti-CD45	Clone 30-F11	Biolegend	103138	AB_2563061
Alexa Fluor 546 conjugated	Anti-DARC	Clone 6B7	Generated in-house		
Alexa Fluor 605 conjugated	Anti-CD31	Clone 390	Biolegend	102427	AB_2563982
	Anti-CD45	Clone 30-F11		103139	AB_2562341

### Confocal microscopy and image analysis

For whole-mount staining, omentum, ear, cremaster muscle, and bladder were harvested from young adult donor mice and immediately fixed in phosphate-buffered 1% paraformaldehyde/1.5% *L*-lysine/0.2% sodium periodate (PLP) sodium phosphate dibasic (0.1 M Na_2_HPO_4_) solution, pH = 7, overnight at 4 °C. Conjunctivae were fixed in acetone for 10 min at room temperature. For frozen sections, tissues were fixed in PLP overnight (4 °C) followed by incubation in 30% sucrose in phosphate-buffered saline (PBS). Samples were snap-frozen in Tissue Freezing Medium (Triangle Biomedical Sciences) and stored at –80 °C. Thin sections (30 μm) were prepared on a cryostat, mounted on Superfrost Plus slides (VWR), and stained with fluorescent antibodies in a humidified chamber after FcR blockade with 2.4G2 antibody. 2.4G2 mAb blocks non-antigen-specific binding of immunoglobulins to the FcγIII and FcγII, and possibly FcγI, receptors. Tissues were stained (overnight, 4 °C) in blocking buffer and washed (see list of buffers in Table 3). Whole-mount preparations were mounted between two coverslips (VWR) with Genteal (Acori); frozen sections were mounted in FluorSave reagent (Calbiochem) and stored at 4 °C.

**Table 3:** Buffer recipes

Buffer name	Recipe
IHC blocking buffer	PBS with 0.5% BSA (Calbiochem) and 0.3% Triton X-100 (Sigma)
IHC washing buffer	PBS with 0.2% BSA (Calbiochem) and 0.1% Triton X-100 (Sigma)
Digestion buffer	HBSS (Corning) with 2% FBS (Gemini), 10 mM HEPES (Corning) and 2 mM CaCl_2_ (Sigma)
FACS buffer	PBS (Lonza) with 5% FCS (Invitrogen-Gibco) and 5 mM EDTA (Boston BioProducts)

Two whole mounts of fresh murine omentum were prepared and stained for CD31 and DARC, as described above, and z-stacks of confocal micrographs were acquired (10 or 15 images at 2.9 μm vertical step intervals). Fluorescent images were used for 3D rendering using Imaris software. Supporting data values for all surface areas are included in Additional file 2.

Confocal images were acquired on an Olympus Fluoview BX50WI inverted microscope with × 10/0.4, ×20/0.5, and × 40/1.3 objectives. Image stacks for three-dimensional reconstructions were acquired at 1–4.5 μm Z-intervals. Image analysis was performed using Volocity (Improvision) or Imaris (Bitplane) software.

### Preparation of aorta

To induce atherosclerosis, Apoe^–/–^ mice were fed a Western diet (Harlan Teklad) for 12 weeks prior to analysis [28]. Aortic root tissue was frozen in OCT and 5-μm thick sections were cut with a cryostat. Sections were fixed in isopropanol for 10 min at 4 °C and blocked with PBS + 10% goat serum + 0.5% BSA. Staining was performed overnight at 4 °C with AF546-anti-DARC antibody + APC-anti-CD31 (MEC 13.3). Samples were fixed in 4% PFA for 10 min, nuclei were stained with Yoyo-1, and sections were mounted using Prolong Gold reagent. Images of the vasa vasorum of aorta were acquired on a Leica SP5 microscope.

### Tissue digestion

Lymph node and brain tissues were digested with 2.5 mg/mL Collagenase D (Roche), 50 μg/mL DNAse I (Roche), and 0.4× protease inhibitor (Roche) in digestion buffer at 37 °C. Digestion of LNs was adapted from Fletcher et al. [29], such that tissues were digested for 20 min without protease inhibitor followed by three 10-min incubations with protease inhibitor. Brain tissues were digested for 15–20 min at 37 °C. Enrichment of ECs was performed by centrifugation at 5000 *g* for 30 min at 4 °C in a dextran solution (17% dextran (Sigma, catalog number 31392)/20 mM HEPES). Skin, colon, and small intestine tissues were digested with 2.5 mg/mL Collagenase D (Roche), 50 μg/mL DNAse I (Roche), and 1× protease inhibitor (Roche) in digestion buffer for 30 min at 37 °C on a rotisserie wheel. Colon and small intestine were washed with 5% FBS and 25 mM HEPES, followed by 2 mM EDTA and 25 mM HEPES, and finally 10% FBS, 5 mM EDTA, and 15 mM HEPES prior to enzymatic digestion to remove epithelial cells. Pancreatic and adipose tissues were digested with 1.25 mg/mL Collagenase D (Roche), 50 μg/mL DNAse I (Roche), and 1× protease inhibitor (Roche) in digestion buffer for 30 min at 37 °C on a rotisserie wheel. Cells were resuspended in FACS buffer (PBS (Lonza), 5% FBS (Invitrogen-Gibco), 5 mM EDTA (Boston BioProducts)) for analysis.

### Flow cytometry

Single-cell suspensions were immunostained, washed, and resuspended in FACS buffer containing 7-AAD viability stain (BioLegend) for immediate acquisition using a BD FACS CANTO, BD LSRII (BD Biosciences), or CytoFlex (Beckman Coulter) and analyzed using FlowJo software (Treestar).

### RT-qPCR

EC subsets were FACS-sorted using an Aria I cell sorter (BD Biosciences) and collected in Trizol (Ambion). RNA extraction was performed prior to cDNA synthesis using the iScript kit (BioRad). qPCR was performed on a LightCycler 480 II (Roche) using SybGreen Quantifast kit (Qiagen). Relative gene expression was calculated using the ∆CT method. See list of primers in Table 1.

### Intravital microscopy (IVM)

IVM of subiliac lymph node, skin, BM, and cremaster muscle was performed as previously described [5, 30–32] using an IV-500 intravital microscope (Mikron Instruments), equipped with a Rapp OptoElectronic SP-20 xenon flash lamp system and QImaging Rolera-MGi EMCCD camera.

### Western blot analysis

Protein extracts were loaded on a 4–12% Tris-Glycine precast gel (Invitrogen). Precision Plus protein Kaleidoscope standards (Bio-Rad, catalog number 161-0375) were used as controls for molecular weight. Heat-denaturation of protein samples was performed. Membranes were incubated with anti-mouse DARC MAb and revealed using HRP-linked anti-rat antibody (Cell Signaling, RRID AB_10694715, catalog number 7077S). Anti-β-actin antibody (Sigma, RRID AB_476743, catalog number A5316) was used for loading control. Immunoreactive proteins were detected with Pierce ECL Western Blotting Substrate (Thermo Scientific).

### Chemokine binding assay

Chemokine binding to RBCs was assessed as previously described [33], with slight modifications. RBCs were incubated with increasing chemokine concentrations (10^–10^–10^–5^ M or 10^–11^–10^–7^ M of CXCL8 or mCXCL1, respectively), with either anti-human DARC MAb Fy6 (gift from Dr. M. Uchikawa, Japanese Red Cross) or anti-mouse DARC MAb. Mean fluorescence of MAb staining was analyzed by FACS. IC_50_ was calculated using GraphPad Prism software.

### Statistical analysis

Results are expressed as means ± SEM. All statistical analyses were performed in Prism (GraphPad Software) or Excel Software. Means between two groups were compared by two-tailed t-test. Means between three or more groups were compared by one-way or two-way ANOVA with Tukey’s or Dunnett’s Multiple Comparison Test. The numbers of replicates or animals are indicated in figure legends.

## Results

### Validation of anti-mouse DARC mAb specificity

**Fig. 1:**
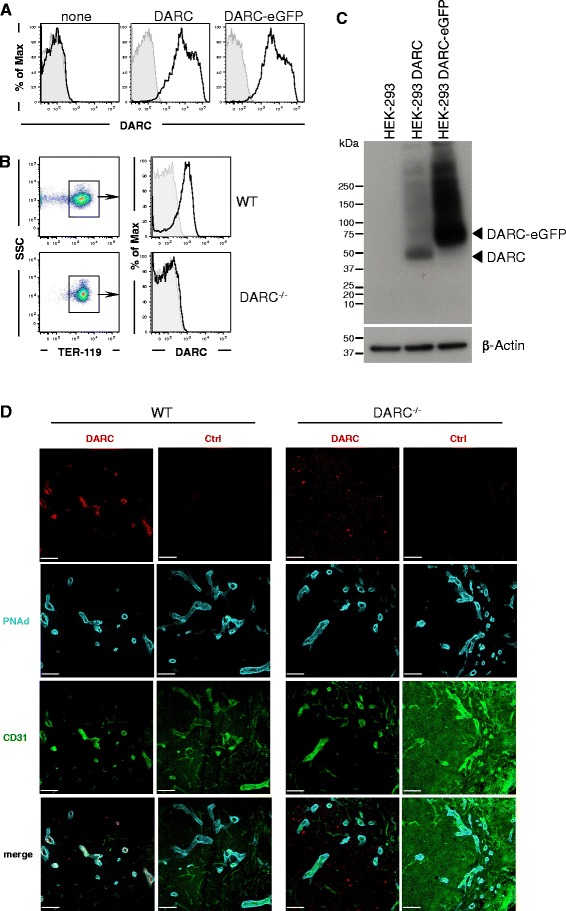
Validation of anti-mouse DARC MAb reactivity. **a** Representative flow cytometry histograms of control HEK-293 cells (none) and HEK-293 cells transfected with DARC or DARC-eGFP fusion protein stained with anti-mouse DARC MAb (n = 3 experiments). **b** Flow cytometry analysis of anti-mouse DARC MAb reactivity with RBCs. Peripheral blood was harvested from wildtype (WT) and DARC^–/–^ mice and stained with a MAb against the RBC marker TER-119 and anti-mouse DARC MAb (*black line*) or isotype control MAb (*grey*) (n > 8 experiments). **c** Western blot analysis of anti-mouse DARC MAb reactivity with protein lysates from untransfected HEK-293 cells and HEK-293 cells stably transfected with DARC or DARC-eGFP fusion protein. b-Actin is shown as loading control (n = 4 experiments). **d** Representative confocal fluorescent micrographs of frozen sections of peripheral lymph nodes from DARC WT and DARC^–/–^ mice stained with MAbs against CD31 (*green*), peripheral node addressin (PNAd; *blue*), and DARC or isotype control (*red*). 20× objective, scale bars = 200 μm (n = 3 experiments)

After immunization of a rat with DARC-eGFP transfected PC-12 cells, hybridoma lines were generated and supernatants screened by FACS. One hybridoma supernatant stained HEK-293 cells transfected with DARC or DARC-eGFP (Fig. 1a), as well as RBCs from C57BL/6 WT mice (Fig. 1b), but not untransfected HEK-293 cells or DARC^–/–^ RBCs. This hybridoma was subcloned and determined to produce a single MAb of IgG2a,k isotype. Both supernatant and purified IgG recognized DARC protein in lysates of DARC or DARC-eGFP transfected HEK-293 cells by Western blot (Fig. 1c). No signal was detected with the parental untransfected HEK-293 line. Robust immunoreactivity was also observed with TER-119^+^ RBCs from Balb/c mice. The MAb did not cross-react with RBCs from other species, including rat and human (Additional file 3: Figure S2). Confocal microscopy of peripheral lymph node (PLN) sections revealed that anti-DARC MAb colocalized with MAb MECA-79, which specifically delineates peripheral node addressin (PNAd), a marker of HEVs [34]. No microvascular reactivity was detected in DARC^–/–^ PLNs (Fig. 1d).

To assess whether the anti-DARC MAb is sensitive to DARC occupancy with chemokines, we assessed staining of WT RBCs in the presence of CXCL1 or CXCL8. Neither chemokine affected binding of the anti-mouse MAb, whereas both blocked staining of human RBCs with anti-human DARC MAb Fy6 (Additional file 3: Figure S2C), consistent with previous studies [35, 36]. Thus, although the exact epitope recognized by our anti-mouse DARC MAb was not identified, it is unlikely to overlap with the chemokine binding region(s) of DARC.

### DARC expression is restricted to V-ECs

**Fig. 2:**
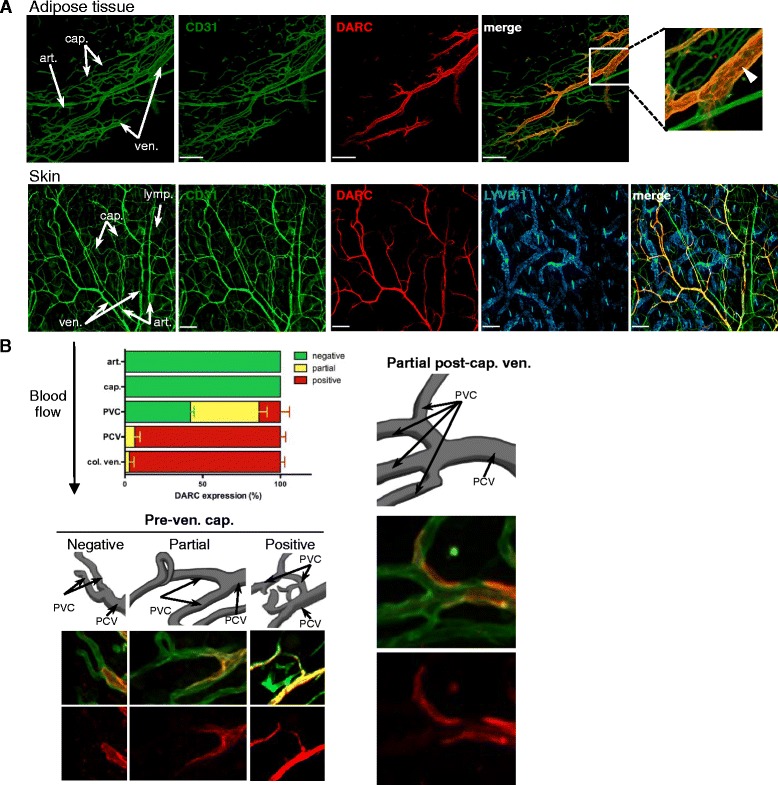
DARC is a robust marker of venular endothelial cell differentiation. **a** Representative confocal micrographs of whole mount stainings of adipose tissue (omentum, 20× objective, scale bars = 200 μm, *top row*) and skin (ear, 10× objective, scale bars = 300 μm, *bottom row*) with MAbs against CD31 (*green*), DARC (*red*), and LYVE-1 (*blue*). *Arrows* in images on the left identify representative arterioles (art.), capillaries (cap.), and venules (ven.). The boxed area in the merged image of adipose tissue was magnified (far right, *top row*) to illustrate concentrated DARC staining at inter-endothelial junctions (*arrowhead*). Adipose tissue n > 10 experiments, skin n > 10 experiments. **b** Semi-quantitative analysis of DARC expression in arterioles (art.), capillaries (cap.), pre-venular capillaries (PVC), post-capillary venules (PCV), and collecting venules (col. ven.) in adipose tissue. Data represent 16 microvascular trees from seven fields of view of omenta from two mice. Supporting data values are included in Additional file 2. Representative confocal micrographs of tissues stained with anti-CD31 (*green*) and anti-DARC (*red*) depict positive, partial, and negative DARC staining on PVCs and partial DARC expression on PCVs. Error bars depict mean ± SEM

Confocal micrographs of whole mounts of murine adipose tissue and skin showed that DARC immunoreactivity was exclusively biased toward venules (Fig. 2a). No signal was detected in either arterioles or the vast majority of capillaries. On V-ECs, DARC appeared to be enriched at intercellular junctions between adjacent ECs.

**Fig. 3:**
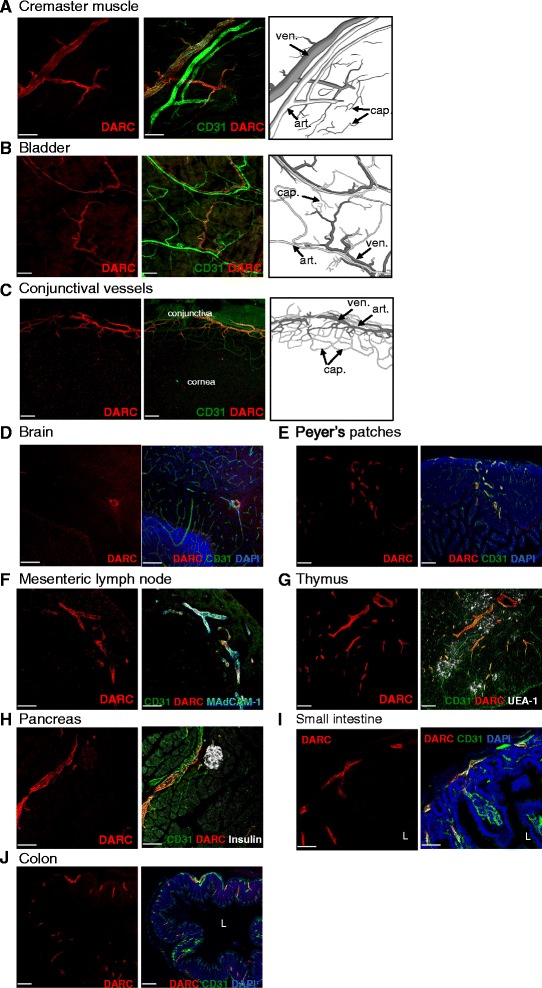
DARC expression on whole mount and frozen section stainings. Representative confocal micrographs of whole mount staining of (**a**) cremaster muscle (20× objective, scale bar = 200 μm), (**b**) bladder (10× objective, scale bar = 300 μm), and (**c**) conjunctival vessels (10× objective, scale bar = 300 μm) with MAbs against CD31 (*green*) and DARC (*red*). Representative confocal micrographs of frozen sections from (**d**) brain (20× objective, scale bar = 200 μm), (**e**) Peyer’s patches (10× objective, scale bar = 300 μm), (**f**) mesenteric lymph nodes (20× objective, scale bar = 200 μm), (**g**) thymus (10× objective, scale bar = 300 μm), (**h**) pancreas (20× objective, scale bar = 200 μm), (**i**) small intestine (20× objective, scale bar = 100 μm), and (**j**) colon (10× objective, scale bar = 300 μm) with MAbs against: CD31 (*green*), DARC (*red*), insulin (*white*), MAdCAM1 (*white*), or UEA-1 (*white*) as indicated. L indicates lumen of small intestine and colon. n = at least three experiments for each tissue

To quantify the frequency of DARC^+^ and DARC^–^ microvessels, we categorized CD31^+^ microvascular segments based on their morphology and caliber in whole mounts of omentum. Virtually all PCVs and CVs were DARC^+^, whereas arterioles and downstream capillaries were consistently DARC^–^. The transition from DARC^–^ to DARC^+^ ECs occurred either at the point where capillaries merged to form a PCV or slightly upstream in pre-venular capillaries (Fig. 2b and Additional file 4: Figure S3). DARC staining in venules was generally uniform and without obvious gaps in immunoreactivity, suggesting that essentially all V-ECs express DARC. Analogous distribution patterns were detected in multiple tissues, including cremaster muscle, bladder, conjunctiva, brain, Peyer’s patches, mesenteric LNs (MLNs), thymus, pancreas, small intestine, and colon (Fig. 3). DARC was undetectable in large veins or arteries, including the lumen of the aorta (Additional file 5: Figure S4 and Additional file 6: Figure S5A). However, DARC was readily detected in vasa vasorum around the aorta of WT mice, and expression in these vessels appeared to be further enhanced in atherosclerotic aortas in *Apoe* ^–/–^ mice (Additional file 6: Figure S5B).

### Flow cytometric analysis of DARC expression on microvascular EC subsets

**Fig. 4:**
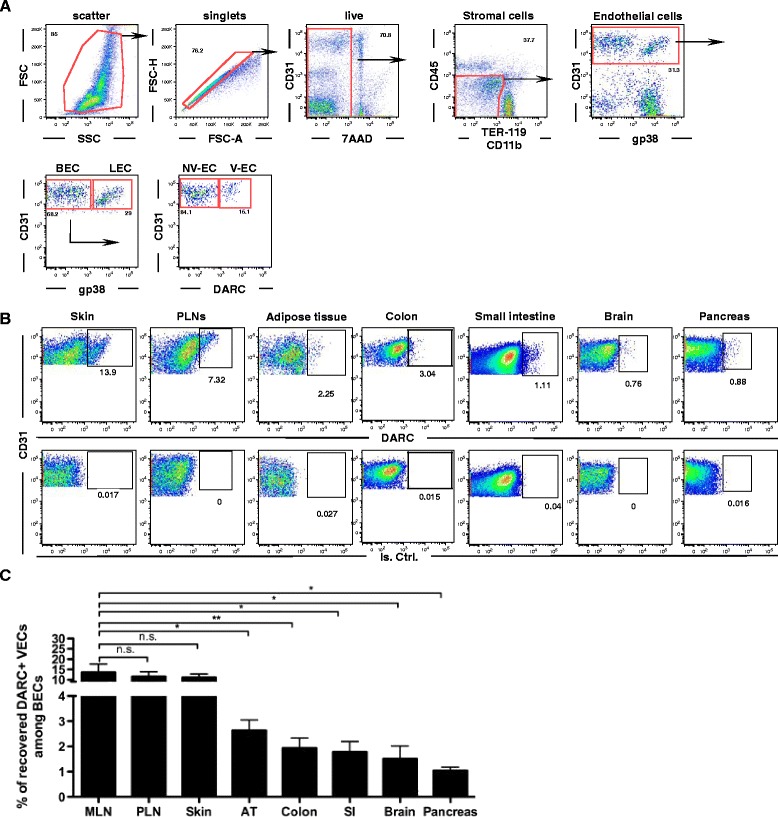
Identification of DARC^+^ venular endothelial cells (V-ECs) by flow cytometry. **a** Gating strategy to identify EC subsets in single cell suspensions of murine lymph nodes; *BEC* blood ECs, *LEC* lymphatic ECs, *NV-EC* non-venular EC. **b** Using the same gating strategy as in panel A, the frequency of DARC^+^ V-EC and DARC^–^ NV-EV among BECs was assessed in multiple tissues (*upper row*). The lower row shows the same samples stained with an isotype-matched control MAb. **c** Quantitative assessment of the frequency of DARC^+^ V-ECs among total BECs in a panel of fresh murine tissues. Error bars depict mean ± SEM of recovered V-EC frequencies among BECs in mesenteric LN (n = 11), peripheral LN (n = 14), skin (n = 18), adipose tissue (n = 6), colon (n = 10), small intestine (n = 6), brain (n = 5), and pancreas (n = 5). ^ns^ *P* > 0.05, *P ≤ 0.05; **P ≤ 0.01

To more precisely define the frequency and distribution of DARC^+^ and DARC^–^ ECs, we stained single-cell suspensions from normal tissues with fluorophore-conjugated anti-mouse DARC MAb combined with markers of leukocytes (CD45), myeloid phagocytes (CD11b), RBCs (TER-119), the pan-endothelial marker CD31, and gp38, which detects lymphatic ECs (LECs). This staining strategy combined with gating on characteristic forward and side light scatter profiles allowed simultaneous discrimination of V-EC (CD45^–^TER-119^–^CD11b^–^gp38^–^CD31^+^DARC^+^), NV-ECs (CD45^–^TER-119^–^CD11b^–^gp38^–^CD31^+^ DARC^–^) and LECs (CD45^–^TER-119^–^CD11b^–^gp38^+^CD31^+^) in multiple tissues (Fig. 4a).

To obtain single-cell suspensions, a number of mouse tissues, including skin, LNs, adipose tissue, colon, small intestine, brain, and pancreas, were mechanically dissociated and incubated with a mixture of proteases. Pilot experiments revealed that the epitope recognized by the anti-mouse DARC MAb on both ECs and RBCs was susceptible to degradation by neutral proteases (Additional file 7: Figure S6). We developed, for each tissue, specific digestion protocols that could reliably preserve DARC staining on both RBCs and V-ECs using distinct combinations of protease cocktails and protease inhibitors (Fig. 4b).

Using this approach, we assessed the frequency of V-ECs among total blood ECs (BECs) in each tissue sample by flow cytometry (Fig. 4c). The recovery of DARC^+^ V-ECs relative to the total yield of BECs was similar in skin and LNs while adipose tissue, colon, small intestine, brain, and pancreas yielded a lower frequency of DARC^+^ V-ECs among BECs. Several factors could potentially contribute to the observed differences between tissues in apparent V-EC frequency, as detected by flow cytometry. (1) V-ECs in some tissues may contain a variably sized population that does not express DARC. However, given the uniformity of venular DARC staining in whole mount preparations, we consider this unlikely. (2) Proteases used to prepare single-cell suspensions for FACS analysis may have destroyed DARC epitopes on a fraction of V-ECs in some tissues, which could potentially lead to an underestimation of the DARC^+^ EC fraction in those tissues. However, our tissue digestion protocols were devised to mitigate this effect as far as possible, and we monitored Ter-119^+^ RBCs in tissue preparations to ensure conservation of DARC epitopes. (3) The relative rate of recovery of V-EC versus NV-EC may differ between tissues. (4) The observed measurements may reflect true differences in V-EC abundance between tissues.

To independently address the latter two (non-exclusive) points, we conducted a digital 3D analysis of a whole mount staining of murine omentum by quantifying the percentage of DARC^+^ venular surface area in the total vascular (CD31^+^) surface area. In the chosen sample, the DARC^+^ fraction was approximately 14% of the total CD31^+^ surface area (Additional file 8: Figure S7). By comparison, the average frequency of DARC^+^ V-ECs among total ECs in adipose tissue was approximately 4%, as detected by flow cytometry (Fig. 4c). This apparent difference must be interpreted with caution for several reasons. Firstly, in order to obtain a sufficient number of ECs from adipose tissue for FACS analysis, we pooled subcutaneous and epididymal fat pads, which might have a lower venular density. Secondly, in order to obtain sufficient resolution of vascular structures of interest within whole-mount preparations, we acquired 3D scans from carefully selected regions that contained, within the same field of view, all microvascular structures of interest, i.e., arterioles, capillary network, and at least one complete venular tree. This limits the choice of suitable areas and may have introduced a selection bias that could result in over-representation of venules. Finally, it is also possible that ECs in different microvascular segments may differ in size. For example, if the average luminal surface area covered per V-EC is greater than that covered by capillary ECs, the venular surface area would be fractionally larger than the percentage of V-ECs. Our current technology does not allow us to accurately resolve individual ECs in intact tissues, so we cannot address this issue with certainty.

Interestingly, a topological analysis of the microvascular anatomy in murine MLNs [37] estimated that HEVs composed approximately 12% of the overall vascular length, which is in good agreement with our flow cytometry data showing 14% DARC^+^ V-ECs among BECs in MLN.

Consistent with reports of DARC expression in human skin [38], murine DARC was detectable by FACS on subsets of LECs in LNs, adipose tissue, and skin (Additional file 9: Figure S8); however, DARC expression on LECs was too low to be detectable in these tissues by confocal microscopy (Figs. 1 and 2).

### DARC as a pan-venular marker

**Fig. 5:**
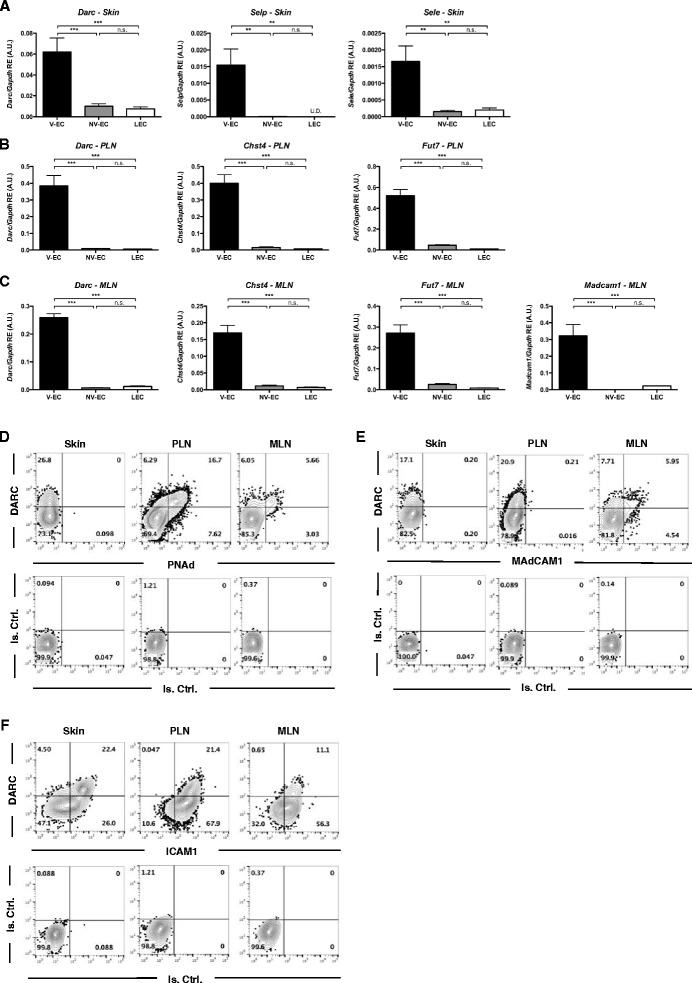
Correlation of DARC expression and known venular markers. **a** RT-qPCR showing DARC (*Darc*), P- (*Selp*), and E-selectins (*Sele*) expression at the mRNA level in cell-sorted V-EC (CD45-CD31 + gp38-DARC+), NV-EC (CD45-CD31 + gp38-DARC-), and LEC (CD45-CD31 + gp38+) from skin tissue. **b** RT-qPCR showing *Darc*, Carbohydrate sulfotransferase 4 (*Chst4*), and Fucosyltransferase VII (*Fut7*) expression at the mRNA level in cell-sorted V-EC, NV-EC, and LEC from peripheral lymph nodes (PLN; pool of inguinals, brachials, axillaries, cervicals, and auriculars). **c** RT-qPCR showing *Darc*, *Chst4*, *Fut7*, and mucosal vascular addressin cell adhesion molecule 1 (*Madcam1*) expression at the mRNA level in cell-sorted V-EC, NV-EC, and LEC from mesenteric lymph nodes (MLN). Expression data are shown relative to GAPDH of a pool of three experiments for each gene and expressed in arbitrary units (A.U.). Error bars depict mean ± SEM. ^ns^ *P* > 0.05, **P ≤ 0.01, ***P ≤ 0.001. Supporting data values are included in Additional file 2. **d**–**f** Flow cytometry contour plots of DARC and peripheral node addressin (PNAd) expression (**d**), DARC and MAdCAM-1 expression (**e**), and DARC and ICAM-1 expression (**f**) in ear skin, PLNs, and MLNs. Cell suspensions were gated on CD45-CD31 + gp38-BEC subset for flow cytometry analysis in all the tissues. n = 3 experiments for each tissue

Next, we asked whether DARC expression correlates with other known venule-associated markers. For example, P- and E-selectin are constitutively expressed (although challenging to detect by immunostaining) in resting skin venules [30, 39, 40]. Indeed, when mRNA levels of *Darc*, *Selp*, and *Sele* were measured by qPCR in sorted dermal DARC^+^ V-ECs, DARC^–^ NV-ECs, and LECs, all three mRNA species were detected exclusively in V-ECs (Fig. 5a).

Similarly, HEVs (unlike other microvessels) in PLN express carbohydrate sulfotransferase 4 (*Chst4*) [41] and fucosyltransferase VII (*Fut7*) [42], enzymes involved in the biosynthesis of 6-sulfo-SLeX on core 1 O-glycans that comprise the PNAd marker [40, 43]. PNAd is also synthesized by most HEVs in MLN, which additionally expresses MAdCAM-1 [44, 45]. Accordingly, *Darc* mRNA expression in PLN and MLN was restricted to V-ECs and correlated closely with *Chst4* and *Fut7* in both tissues and with *Madcam1* in MLNs (Fig. 5b, c). These observations were confirmed at the protein level by FACS analysis of PLN and MLN BECs. Of note, while almost all PNAd^+^ and MAdCAM-1^+^ BECs expressed DARC, up to approximately 15% of DARC^+^ ECs were PNAd^–^ and MAdCAM-1^–^ (Fig. 5d, e). These ECs likely derived from medullary segments of the venular tree, which are lined by flat ECs and do not express vascular addressins [46].

Since PNAd, MAdCAM-1, and the endothelial selectins are not only restricted to V-ECs, but are also subject to a strong tissue bias at steady-state [47], we also stained BECs for ICAM-1, which is constitutively expressed on ECs throughout the body [48]. Indeed, BECs expressed ICAM-1 in both skin and LNs, whereas PNAd and MAdCAM-1 were absent on dermal BECs [49] (Fig. 5d–f). Interestingly, although ICAM-1 expression was more pronounced and uniform in venules than on other ECs, it was also expressed, albeit at a lower level, on most DARC^–^ NV-ECs. Thus, when compared to other known venular surface moieties, DARC stands out as a truly pan-venular marker that shows exquisite specificity for V-ECs and is readily detectable by IHS and flow cytometry in every murine tissue analyzed.

### Detection of venular DARC expression by IVM

**Fig. 6:**
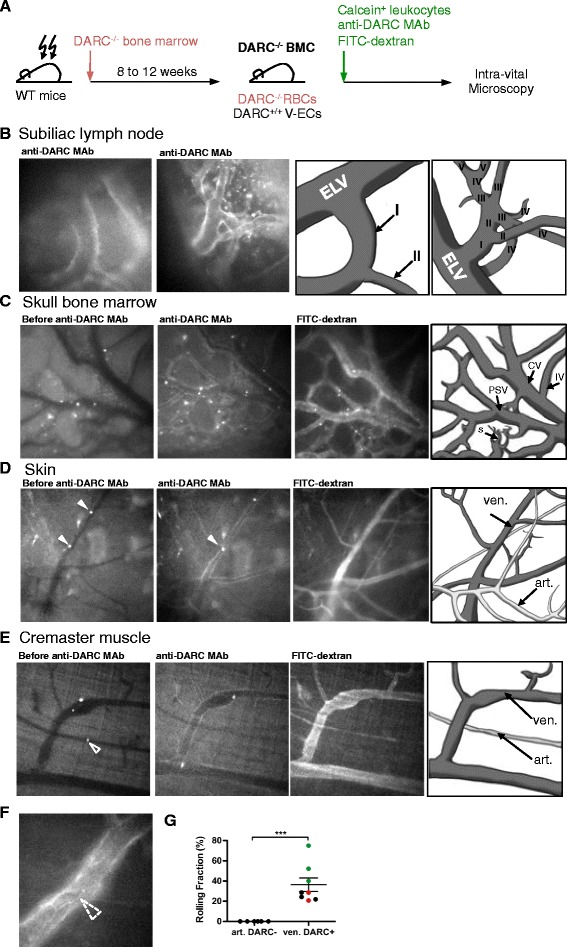
Intravital microscopy (IVM) of leukocyte adhesion in DARC^+^ venules. **a** Schematic representation of IVM experiment. **b** Alexa Fluor 488 labeled anti-mouse DARC MAb staining of microvessels in subiliac lymph node. Branching order designation of intranodal venular segments was performed as in ref. [5]. Left: micrograph at the LN hilus showing a large DARC^+^ CV (order I) that drains venous blood into a DARC^–^ extra-lymphoid vein (ELV); 20× objective. Right: Representative intranodal venular tree with branching orders identified in Roman numerals. **c** DARC expression in BM intermediate venules (IV), collecting venules (CV), post-sinusoids venules (PSV), and sinusoids (s). Microvascular segment designation was as in [31]; 10× objective (n = 3 experiments). **d** Intravital fluorescent micrographs of murine ear skin after injection of fluorescent leukocytes. Images were taken during an initial control period (*left*), after injection of Alexa Fluor 488 labeled anti-mouse DARC MAb (*middle*) and after subsequent injection of FITC-dextran, which delineates the intravascular compartment in all microvessels. Leukocytes rolling in DARC^+^ venules are identified by arrowheads. Arteriole (art.), venule (ven.); 20× objective (n = 3 experiments). See Additional file 10: movie 1. **e** Leukocyte rolling in DARC^+^ venules in cremaster muscle; 10× objective. Arrowhead in left panel indicates a non-adherent leukocyte captured while passing through an arteriole. See Additional file 11: movie 2. **f** *Dashed arrowhead* indicates pronounced DARC expression at the junction between adjacent ECs in cremaster muscle; 40× objective. **g** Rolling fractions (percent of rolling cells in the total flux of cells passing a microvessel) of calcein-labeled neutrophils in ear skin microvessels was assessed; n = 3 mice, data points from same animal are highlighted in same color (*black*, *red* or *green*). Error bars show mean ± SEM. ***P < 0.001. Supporting data values for this analysis are included in Additional file 2

Having determined that our anti-DARC MAb recognizes surface-expressed DARC in frozen sections and whole mount preparations of murine tissues ex vivo and on intact transfectants and isolated ECs in vitro, we asked whether the MAb could pinpoint microvascular DARC expression in vivo. We transplanted irradiated C57BL/6 mice with DARC^–/–^ BM (Fig. 6a); at 8 weeks after BM transplantation, this resulted in absent DARC expression on RBCs. Thus, upon intra-arterial injection of Alexa Fluor-488 labeled anti-DARC, the MAb remained free to bind endothelial DARC without being captured by DARC^+^ RBCs.

Luminal DARC expression was readily detectable by epifluorescence IVM [5] in HEVs and large collecting venules in subiliac LNs (Fig. 6b). Epifluorescence IVM of intact skull BM [31] identified prominent DARC expression in intermediate venules, collecting venules, post-sinusoidal venules, and sinusoids (Fig. 6c). Segmental microvascular immunoreactivity, particularly at inter-cellular junctions was also readily detected by IVM in intact ear skin and in surgically exposed cremaster muscle (Fig. 6d–f). By contrast, as expected, none of these IVM models showed detectable DARC expression in arterioles or capillaries.

### DARC^+^ microvessels uniquely support physiologic leukocyte adhesion

To independently verify that the DARC^+^ microvessels detected by IVM were indeed venules, we made use of the fact that leukocytes undergo constitutive selectin-mediated rolling interactions in venules, but not in capillaries or arterioles in murine ear skin [30]. Thus, we injected fluorescent anti-DARC MAb into mice and then injected fluorescently tagged leukocytes into the carotid artery to quantify leukocyte rolling fractions in dermal DARC^+^ and DARC^–^ microvessels by IVM [30]. All DARC^+^ microvessels supported robust rolling interactions, whereas rolling was absent in all DARC^–^ microvessels, indicating that DARC delineates specifically pro-adhesive venules, but not non-adhesive non-venular microvessels (Fig.  6 g, Additional file 10: movie 1).



**Additional file 10: movie 1.** DARC^+^ microvessels uniquely support physiological leukocyte adhesion in skin. DARC^–/–^ bone marrow chimeric mice were anaesthetized and ear skin was positioned on a custom-built stage for epifluorescence intravital microscopy. BM neutrophils were enriched using a 60–72% Percoll gradient and labeled with calcein AM (5 μM) prior to injection through a catheter in the carotid artery. Subsequently, Alexa Fluor 488 anti-DARC MAb (50–100 μg/mouse) was injected and cell behavior in venules was recorded under stroboscopic epi-illumination in 10 min recordings through 20× water-immersion objectives. Injection of FITC-Dextran (2 mDa) delineates the intravascular compartment in all microvessels. All DARC^+^ microvessels supported robust rolling interactions, whereas rolling was absent in all DARC^–^ microvessels, indicating that DARC delineates specifically pro-adhesive venules, but not non-adhesive non-venular microvessels. (MOV 9903 kb)




**Additional file 11: movie 2.** DARC^+^ microvessels uniquely support physiological leukocyte adhesion in cremaster muscle. DARC^–/–^ bone marrow chimeric mice were anaesthetized and cremaster muscle was positioned on a custom-built stage for epifluorescence intravital microscopy. BM neutrophils were enriched using a 60–72% Percoll gradient and labeled with calcein AM (5 μM) prior to injection through a catheter in a femoral artery feeding the tissue. Subsequently, Alexa Fluor 488 anti-DARC MAb (50–100 μg/mouse) was injected and cell behavior in venules was recorded under stroboscopic epi-illumination in 10 min recordings through 10× water-immersion objectives. Injection of FITC-Dextran (2 mDa) delineates the intravascular compartment in all microvessels. All DARC^+^ microvessels supported robust rolling interactions, whereas rolling was absent in all DARC^–^ microvessels, indicating that DARC delineates specifically pro-adhesive venules, but not non-adhesive non-venular microvessels. (MOV 16573 kb)


## Discussion

Julius Cohnheim first noted that leukocyte recruitment from blood to inflamed tissues occurs preferentially within venular segments of the microcirculation [50]. Indeed, numerous studies have confirmed this fundamental observation in multiple tissues and a variety of species [6, 51, 52]. However, this property of venules is not explained by hemodynamic factors, but a consequence of endothelial specialization [3]. To date, the molecular ‘wiring diagram’ that enables V-ECs to function as the principal gatekeepers for leukocyte access to tissues remains unknown.

Notwithstanding, a handful of venular surface molecules are known to be restricted to a specific vascular bed. Vascular addressins, PNAd [34] and MAdCAM-1 [44], in HEVs support leukocyte recruitment to secondary lymphoid organs. P- and E-selectin mediate leukocyte rolling at steady-state in skin [49], BM [31, 53], and thymus [54], as well as other organs during acute inflammation [55, 56]. Venules may also be distinguished from arterioles by higher vWF expression, although this difference is subtle and does not always allow unequivocal determination of microvascular identity [57]. Indeed, no single marker is known to date to robustly and unequivocally discriminate between venular and non-venular endothelium in all (or most) tissues. The existence of such a marker and an appropriate biological tool to detect that marker on intact ECs could facilitate further studies to uncover the molecular underpinnings of V-EC and NV-EC phenotype and function.

Here, we explored whether a newly developed anti-mouse DARC MAb is useful to discriminate and isolate primary V-ECs and NV-ECs in laboratory mice. Several studies have suggested that DARC positively and selectively identifies V-ECs, at least in some human [13, 14] and murine tissues [15]. However, a rigorous analysis of the macro- and microanatomic distribution of DARC has not been performed, presumably because previously available reagents did not have sufficient sensitivity and/or specificity. This is not surprising considering that DARC is highly conserved among mammals; the nucleotide sequences for mouse (NM_010045.2) and rat (XM_002728033.3) DARC share an overall identity of 88%. When comparing protein sequences, it must be noted that DARC is a serpentine receptor with four variably sized extracellular domains separated by seven transmembrane domains [58]. For DARC detection on intact cells, antibodies must recognize extracellular epitopes on the native receptor. The ectodomains of DARC in mouse and rat share 85.2% similarity in the N-terminal ectodomain and 100%, 65%, and 86.3% identity in ectodomains 1, 2, and 3, respectively. The overall extracellular sequence identity in mice and rats is 83.76%, corresponding to 19 amino acids that differ between the two species to serve as potential epitope(s) for non-self-reactive antibodies.

Despite this high homology between mouse and rat DARC, we were able to immunize rats using a stably transfected rat cell line that over-expressed mouse DARC as the immunogen. This strategy yielded a MAb that specifically and selectively recognized DARC on transfectants, erythroid cells, and microvascular ECs. This reagent clearly delineated DARC^+^ venules in lymphoid and non-lymphoid tissues of different mouse strains in vitro and in vivo. The MAb was active in multiple applications, including immunofluorescence staining of whole mounts and tissue sections, FACS, western blot, and IVM. After i.v. injection of fluorescent anti-DARC MAb, selective staining of V-ECs was readily detectable by IVM in four different tissues. In each setting, circulating leukocytes interacted exclusively with DARC^+^ microvessels, indicating that our MAb positively identifies microvessels capable of supporting leukocyte trafficking.

Our analysis of restricted venular DARC expression is consistent with previous reports that have shown DARC^+^ V-ECs in PLNs, MLNs, and brain [15, 17]. In addition, we report that DARC is also a venule-specific marker in many other murine tissues that had not been examined previously, including the skin, BM, adipose tissue, cremaster muscle, bladder, conjunctival vessels, small intestine, colon, Peyer’s patches, thymus, and pancreas. Of note, DARC expression has also been reported on Purkinje cells [22]. However, although the polyclonal serum of the immunized rat stained Purkinje cells in frozen sections, our anti-mouse DARC MAb failed to do so. The reason for this differential immunoreactivity is unclear, but might reflect differential post-translational modification(s) of Purkinje cell-expressed DARC at or near the epitope recognized by the MAb. Consistent with this idea, our western blot analysis showed a discrepancy between the predicted molecular mass of mouse DARC (36 kDa) and the apparent molecular weight (~45 kDa) of cell-derived protein. DARC has multiple potential sites for post-translational modifications, such as glycosylation, phosphorylation, and tyrosine sulfation, which have been described for other chemokine receptors. Indeed, human DARC is glycosylated in positions 16 and 33 [59], but it has not been determined whether DARC may undergo tissue-specific post-translational modifications.

The fact that our anti-DARC MAb is compatible with multi-color flow cytometry to discriminate between different subsets of primary microvascular ECs allowed us to compare the composition of vascular beds from different tissues. Our analysis suggests that the skin and LNs contain a larger fraction of DARC^+^ V-ECs than most other tissues, which could potentially reflect the fact that these organs undergo particularly vigorous immunosurveillance and are frequent staging grounds for immune responses against invading pathogens, requiring a large vascular surface area capable of supporting leukocyte trafficking. However, it should be cautioned that the recovery of intact EC subsets from tissue preparations is likely incomplete and may not necessarily reflect the true abundance of ECs in situ. Moreover, we observed that the epitope recognized by our MAb is sensitive to proteolytic degradation during tissue digestion. Thus, to achieve reliable yields of DARC^+^ V-ECs and DARC^–^ NV-ECs, it was necessary to develop customized isolation protocols for each tissue, which could potentially impact the apparent abundance of V-ECs in any given tissue.

Erythrocyte-expressed DARC binds inflammatory chemokines and, in doing so, RBCs serve as a sink or reservoir modulating circulating chemokine abundance and pharmacokinetics [25, 60]. DARC expression during murine and human erythroid development has been previously examined only at the mRNA level [36] and by western blot of in vitro cultured erythroblasts [35]. In contrast to these analytical methods, although DARC was readily detectable on RBCs in peripheral blood, DARC expression is even more pronounced on BM-resident erythroid progenitors [61]. Thus, it will be important to investigate the role of erythroid and endothelial DARC in BM during hematopoiesis. In addition, DARC was detectable in BM sinusoidal ECs but was absent from BM-resident myeloid cells. These results differ from a recent study that reported that a polyclonal anti-DARC Ab stained a subset of macrophages, but not ECs, in single-cell suspensions of murine BM [24]. These contrasting observations remain to be fully explained, but could potentially reflect differential reactivity of the immunoreagents used or distinct DARC epitope availability on specific host cell types, or unrecognized loss of DARC epitope(s) during tissue preparation, or any combination of these factors.

The physiological role of DARC is rooted in its capacity to bind a variety of inflammatory chemokines [10, 11]. Several DARC ‘knockout’ mouse strains have been generated whose phenotype indicates a regulatory role for DARC on ECs and RBCs during inflammatory processes [25, 62, 63]. When inflammatory chemokines are generated in the extravascular space, they bind DARC on the basolateral side of V-ECs and the complex is then transcytosed across the caveoli network to the luminal surface for presentation to passing leukocytes [12]. The tight restriction of DARC expression to small venules focusses chemokine bioavailability to postcapillary microvessels and hence is likely to contribute to the strong bias of leukocyte migration in venular segments. Of note, immunofluorescence staining of whole-mount tissues suggests that DARC is concentrated at intercellular junctions between V-ECs, similar to other leukocyte traffic molecules such as CD31 [64, 65], ICAM-2 [66, 67], and CXCR7 [68]. The physiological significance of this observation remains undetermined.

Interestingly, although DARC expression was uniform and prominent in PCVs and in small to mid-size CVs in every murine tissue examined, it was absent in larger CVs and veins, indicating that DARC is not merely a marker of ECs exposed to deoxygenated blood. In fact, the environmental signal(s) that regulate(s) microvascular DARC expression remain(s) to be identified. It is also not clear whether all ECs that interact with blood-borne leukocytes inevitably express DARC. In fact, our results suggest that this is unlikely since atherosclerotic plaques in aortas of *Apoe* ^–/–^ mice did not display luminal DARC. However, DARC^+^ microvessels were readily identified within the aortic wall in C57BL/6 mice, and DARC^+^ vasa vasorum were even more prominent in aortas of *Apoe* ^–/–^ mice. Thus, a network of microvessels that include *bona fide* venules supplies large arteries prone to atherosclerosis in mice. Whether and to what extent these vessels can contribute to the recruitment of atherogenic leukocytes remains to be determined.

## Conclusion

In summary, we report the generation and extensive characterization of a MAb against mouse DARC/ACKR1/CD234. Using this new biological tool, we demonstrate that DARC is truly a pan-venular marker. This MAb will be useful for future studies on the cellular and molecular underpinnings of segmental endothelial specialization in the peripheral microvasculature.

## Additional files

Additional file 1: Figure S1. Generation of monoclonal anti-mouse DARC antibody. To generate the monoclonal anti-mouse DARC antibody, rats were immunized with a stably transfected PC-12 rat cell line expressing DARC-eGFP fusion protein. (A) Validation of membrane expression was performed by confocal microscopy using HEK-293 cells stably transfected to express DARC-eGFP. (B) Before immunization, DARC-eGFP PC-12 cells were treated with sodium butyrate to maximize the expression of the transfected fusion protein. Flow cytometry histogram shows eGFP fluorescence intensity on PC-12 cells before (grey) and after 24 hours of sodium butyrate treatment (black). (C) Adult rats were immunized four times at 2-week intervals with 5 × 10^6^ sodium butyrate conditioned DARC-eGFP PC-12 transfectants. The first immunization was performed with Complete Freund Adjuvant via s.c. and i.p. routes, the second injection was performed with Incomplete Freund Adjuvant i.p., and the third and last injections were each administered i.p. without adjuvant. (D) Immune sera were screened by flow cytometry for reactivity with DARC ectodomains using HEK-293 cells expressing DARC-eGFP fusion protein. Fluorescence intensity is expressed as geometric mean of fluorescence (GeoMFI). Following splenocyte fusion, twelve 96-well plates were screened by flow cytometry, only two wells showed reactivity against mouse DARC. One clone producing an anti-mouse DARC MAb was isolated, expanded, subcloned, purified, and labeled for this study. (PDF 190 kb)

Additional file 2: Raw data for Fig 2b, Fig 5, Fig 6g, Additional file 7: Figure S6B and Additional file 8: Figure S7. (XLS 217 kb)

Additional file 3: Figure S2. Anti-mouse DARC MAb cross-reactivity and function. (A) Representative flow cytometry histograms of TER-119^+^ RBCs and CD45^+^ hematopoietic cells stained with anti-mouse DARC MAb (black) and isotype control (grey) from C57BL/6 and BALB/c mice (n = 6 mice per group). (B) Representative flow cytometry histograms of mouse, rat, and human RBCs stained with anti-mouse DARC MAb (black) and isotype control (grey). The anti-mouse DARC MAb does not show specific reactivity for the rat and human erythrocyte form of DARC protein (n = 2 individuals per group), (C) Blood was taken from Duffy-positive laboratory donors and 10^6^ red cells were incubated with increasing concentrations of CXCL8 and mCXCL1 in 100 μL PBS with 0.5% BSA for 1 h at 37 °C and subsequently 1 μL of anti-human Fy6 for 30 min, and finally 1 μL of PE-conjugated goat anti-mouse antibody added. For determination of inhibition of directly conjugated anti-murine DARC antibody binding by chemokines, blood was taken from wildtype mice and 10^6^ red cells were incubated with increasing concentrations of CXCL8 and mCXCL1 in 100 μL PBS with 0.5% BSA for 1 h at 37 °C and subsequently 1 μL of Alexa-647 conjugated anti-murine DARC for 30 min. Mean fluorescence of DARC MAb stainings were measured by flow cytometry. (PDF 218 kb)

Additional file 4: Figure S3. Quantification of DARC expression on blood microvasculature. To determine DARC expression on arterioles, capillaries, pre-venular capillaries (PVC), post-capillary venules (PCV), and collecting venules, we analyzed DARC expression in a microvascular network stained with anti-CD31 (green) and anti-DARC (red). White squares indicate the regions selected to illustrate positive, partial, or negative pre-venular capillaries (PVC) for DARC expression as well as partial DARC expression on post-capillary venules (PCV) in Fig. 2; 20× objective, scale bars = 200 μm. (PDF 391 kb)

Additional file 5: Figure S4. DARC expression on vein and artery. Representative confocal micrographs of whole mount staining of femoral vessels stained with anti-DARC or isotype control (red), anti-CD31 (green), and DAPI (blue) as indicated. Bright field indicates the localization of vein and artery. DARC is not detected on vein and artery but is expressed on venules (arrowhead) in the microvasculature of the surrounding connective tissue; 10× objective, scale bars = 300 μm (n = 3 experiments). (PDF 731 kb)

Additional file 6: Figure S5. DARC positive vessels in vasa vasorum of aorta of wildtype (WT) and *Apoe* ^–/–^ mice. Representative confocal micrographs of DARC expression on venules in the vasa vasorum of aorta of WT (A) or *Apoe* ^–/–^ mice (B). Tissues were stained with anti-CD31 (green), anti-DARC MAb (red), and Yoyo-1 (blue) was used to stain nuclei. Arrows indicate DARC^+^ vessels and arrowheads indicate DARC^–^ vessels (WT n = 5 experiments, Apoe^–/–^ n = 8 experiments). (PDF 426 kb)

Additional file 7: Figure S6. DARC epitope sensitivity to enzymatic digestion. (A) Representative flow cytometry histograms of DARC expression on RBCs. RBCs from blood were digested at 37 °C with different concentration of enzymes as indicated: Collagenase VII (1×) = 86 μg/mL, Collagenase D (1×) = 2.5 mg/mL and Collagenase II (1×) = 1.5 mg/mL. RBCs at 4 °C and 37 °C without digestion are used as positive controls for DARC expression. Flow cytometry was performed to detect DARC expression (n = 5 experiments). (B) Quantification of DARC expression on RBCs, results are shown as delta Geometric Mean Intensity of Fluorescence (n = 3 mice/group). Error bars show mean ± SEM. ^ns^ *P* > 0.05, **P ≤ 0.01, ***P ≤ 0.001. Supporting data values are included in Additional file 2. (PDF 180 kb)

Additional file 8: Figure S7. DARC expression in whole mount adipose tissue. Confocal micrographs of whole mount staining of adipose tissue (omentum) were analyzed for CD31 (green) and DARC (red) expression. The fluorescence intensity images (upper panels) and the corresponding 3D rendering images (lower panels) are shown. Surface area for CD31 and DARC channels were calculated using Imaris software and shown as μm^2^. 10× objective, scale bars = 100 μm. Supporting data values are included in Additional file 2. (PDF 261 kb)

Additional file 9: Figure S8. DARC expression on lymphatic endothelial cells. (A) Flow cytometry analysis of DARC expression on lymphatic endothelial cells (LECs) in mesenteric lymph node (MLN), peripheral lymph node (PLN), adipose tissue, and skin. Live LECs are defined as followed: CD45 negative, CD31 and gp38 positive. DARC expression is shown as frequency of LEC subset. (B) Bar graph showing the frequency of DARC^+^ LECs among total LEC population in MLN (n = 7), PLN (n = 6), adipose tissue (AT) (n = 6), and skin (n = 6). Data were excluded from the analysis if number of events in LEC gate was less than 500. Error bars show mean ± SEM. *P ≤ 0.05, ***P ≤ 0.001. (C) DARC expression shown as geometric mean of intensity of fluorescence (Geo.MFI) on venules (V-EC), lymphatic EC (LEC), and non-venules (NV-EC). Error bars show mean ± SEM. ^ns^ *P* > 0.05, **P ≤ 0.01, ***P ≤ 0.001. “#” Identical statistic results for skin, MLN, and PLN. Statistic results for AT are indicated in blue. (PDF 186 kb)

## Abbreviations

BEC: blood endothelial cell
BM: bone marrow
CV: collecting venule
DARC/ACKR1: duffy antigen receptor for chemokines/atypical chemokine receptor 1
EC: endothelial cell
HEV: high endothelial venule
IVM: intravital microscopy
LEC: lymphatic endothelial cell
LN: lymph node
MLN: mesenteric LNs
NV-EC: non venular endothelial cell
PCV: post-capillary venule
PLN: peripheral lymph node
PNAd: peripheral node addressin
RBC: red blood cells
V-EC: venular endothelial cell
WT: wildtype
