# Supplementary figures and images for: Differential DARC/ACKR1 expression distinguishes venular from non-venular endothelial cells in murine tissues
Source: BMC Biol. 2017 May 19;15:45. doi: 10.1186/s12915-017-0381-7 (PMC5438556; doi:10.1186/s12915-017-0381-7)

## Supplemental Figure 1: Generation of the monoclonal anti-mouse DARC antibody

**A**

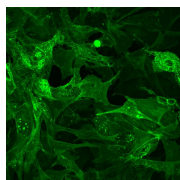

**B**

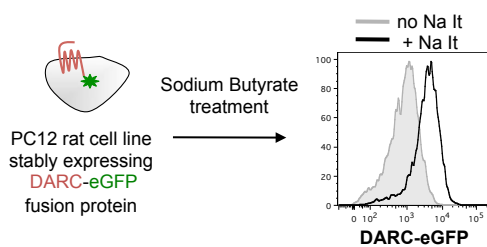

**C**

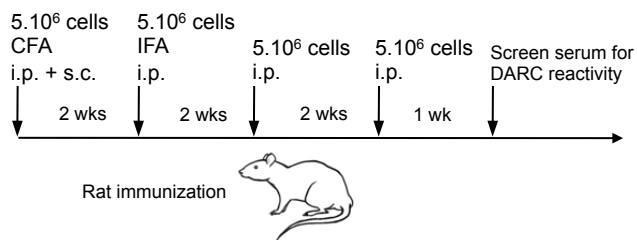

**D**

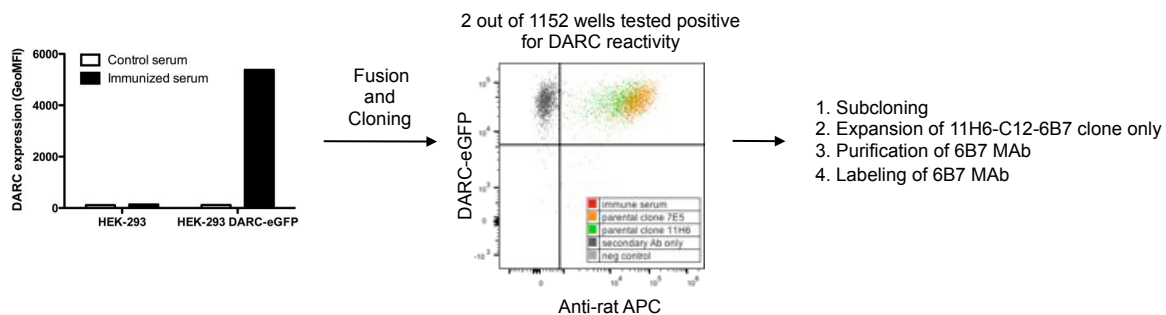

Supplement: Supplementary file 3 — Generation of monoclonal anti-mouse DARC antibody. To generate the monoclonal anti-mouse DARC antibody, rats were immunized with a stably transfected PC-12 rat cell line expressing DARC-eGFP fusion protein. (A) Validation of membrane expression was performed by confocal microscopy using HEK-293 cells stably transfected to express DARC-eGFP. (B) Before immunization, DARC-eGFP PC-12 cells were treated with sodium butyrate to maximize the expression of the transfected fusion protein. Flow cytometry histogram shows eGFP fluorescence intensity on PC-12 cells before (grey) and after 24 hours of sodium butyrate treatment (black). (C) Adult rats were immunized four times at 2-week intervals with 5 × 10^6^ sodium butyrate conditioned DARC-eGFP PC-12 transfectants. The first immunization was performed with Complete Freund Adjuvant via s.c. and i.p. routes, the second injection was performed with Incomplete Freund Adjuvant i.p., and the third and last injections were each administered i.p. without adjuvant. (D) Immune sera were screened by flow cytometry for reactivity with DARC ectodomains using HEK-293 cells expressing DARC-eGFP fusion protein. Fluorescence intensity is expressed as geometric mean of fluorescence (GeoMFI). Following splenocyte fusion, twelve 96-well plates were screened by flow cytometry, only two wells showed reactivity against mouse DARC. One clone producing an anti-mouse DARC MAb was isolated, expanded, subcloned, purified, and labeled for this study. (PDF 190 kb) [file 12915_2017_381_MOESM1_ESM.pdf]

Supplemental Figure 2: Anti-mouse DARC MAb cross-reactivity and function.

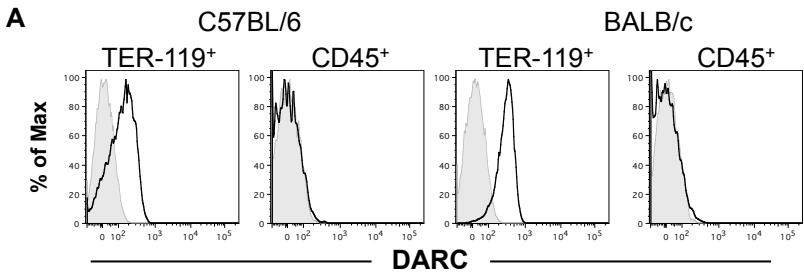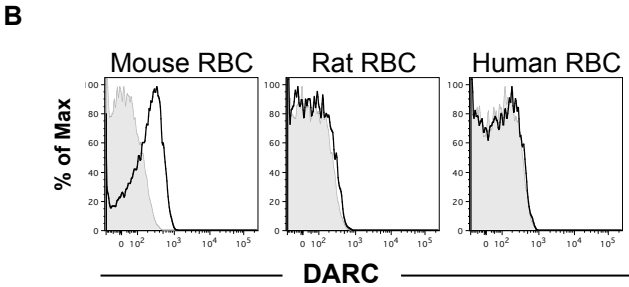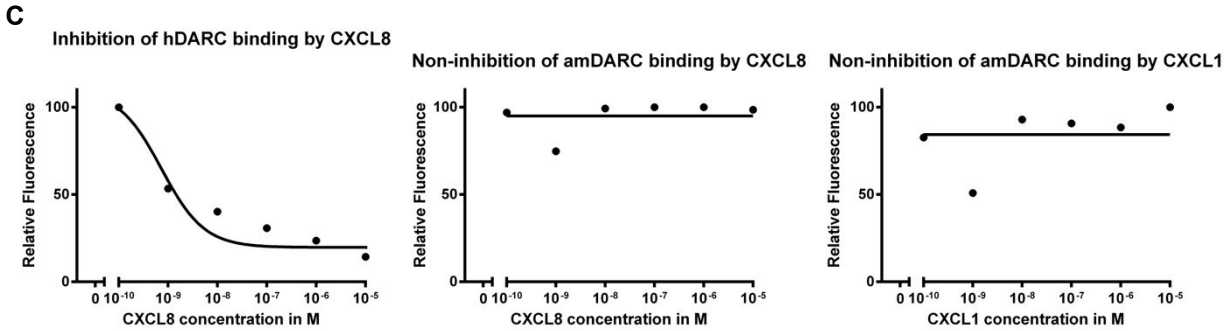

Supplement: Supplementary file 5 — Anti-mouse DARC MAb cross-reactivity and function. (A) Representative flow cytometry histograms of TER-119+ RBCs and CD45+ hematopoietic cells stained with anti-mouse DARC MAb (black) and isotype control (grey) from C57BL/6 and BALB/c mice (n = 6 mice per group). (B) Representative flow cytometry histograms of mouse, rat, and human RBCs stained with anti-mouse DARC MAb (black) and isotype control (grey). The anti-mouse DARC MAb does not show specific reactivity for the rat and human erythrocyte form of DARC protein (n = 2 individuals per group), (C) Blood was taken from Duffy-positive laboratory donors and 106 red cells were incubated with increasing concentrations of CXCL8 and mCXCL1 in 100 μL PBS with 0.5% BSA for 1 h at 37 °C and subsequently 1 μL of anti-human Fy6 for 30 min, and finally 1 μL of PE-conjugated goat anti-mouse antibody added. For determination of inhibition of directly conjugated anti-murine DARC antibody binding by chemokines, blood was taken from wildtype mice and 106 red cells were incubated with increasing concentrations of CXCL8 and mCXCL1 in 100 μL PBS with 0.5% BSA for 1 h at 37 °C and subsequently 1 μL of Alexa-647 conjugated anti-murine DARC for 30 min. Mean fluorescence of DARC MAb stainings were measured by flow cytometry. (PDF 218 kb) [file 12915_2017_381_MOESM3_ESM.pdf]

Supplemental Figure 4: DARC expression on vein and artery

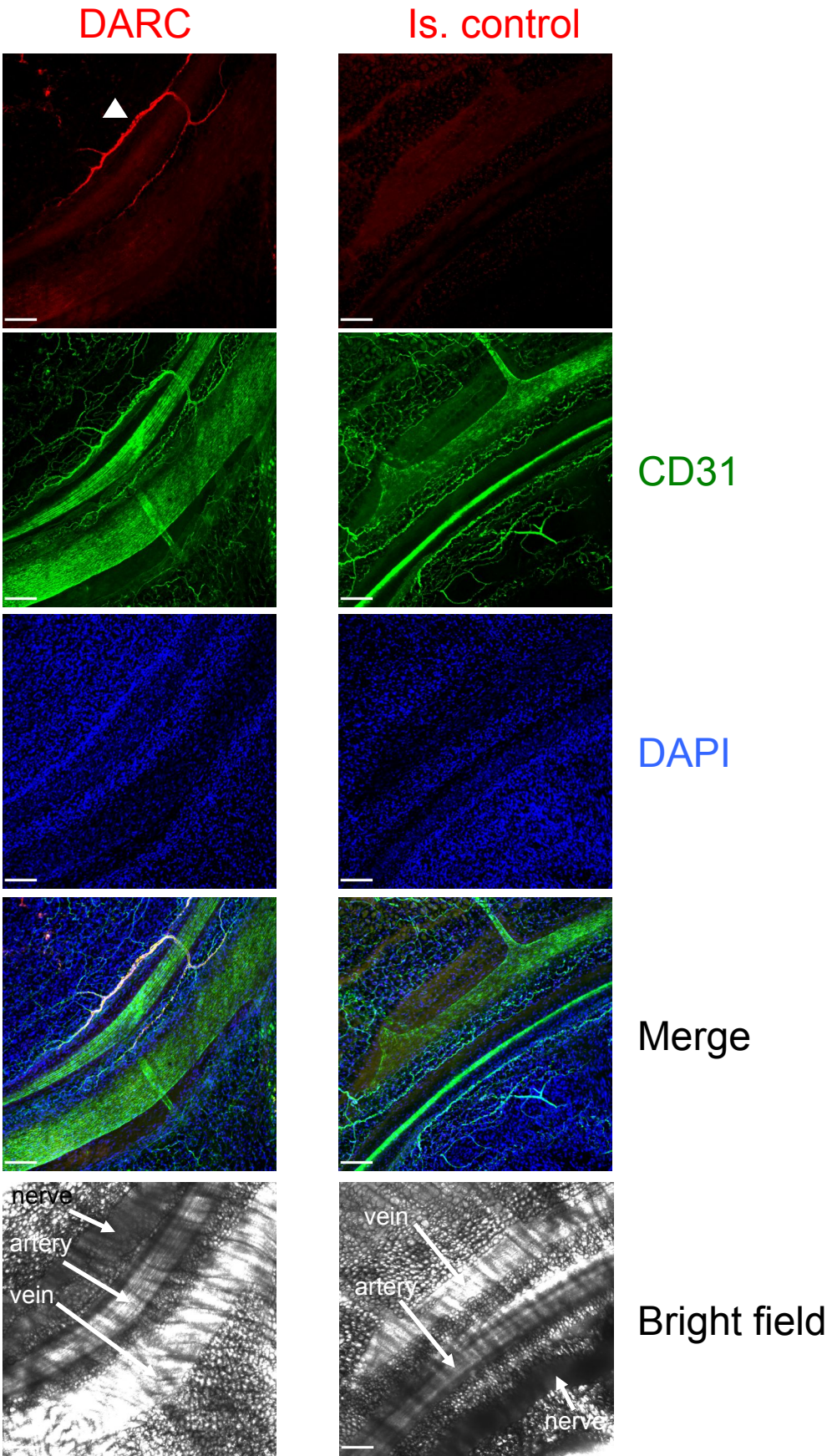

Supplement: Supplementary file 7 — DARC expression on vein and artery. Representative confocal micrographs of whole mount staining of femoral vessels stained with anti-DARC or isotype control (red), anti-CD31 (green), and DAPI (blue) as indicated. Bright field indicates the localization of vein and artery. DARC is not detected on vein and artery but is expressed on venules (arrowhead) in the microvasculature of the surrounding connective tissue; 10× objective, scale bars = 300 μm (n = 3 experiments). (PDF 731 kb) [file 12915_2017_381_MOESM5_ESM.pdf]

Supplemental Figure 5: DARC positive vessels in vasa vasorum of aorta in WT and *Apoe*<sup>-/-</sup> mice

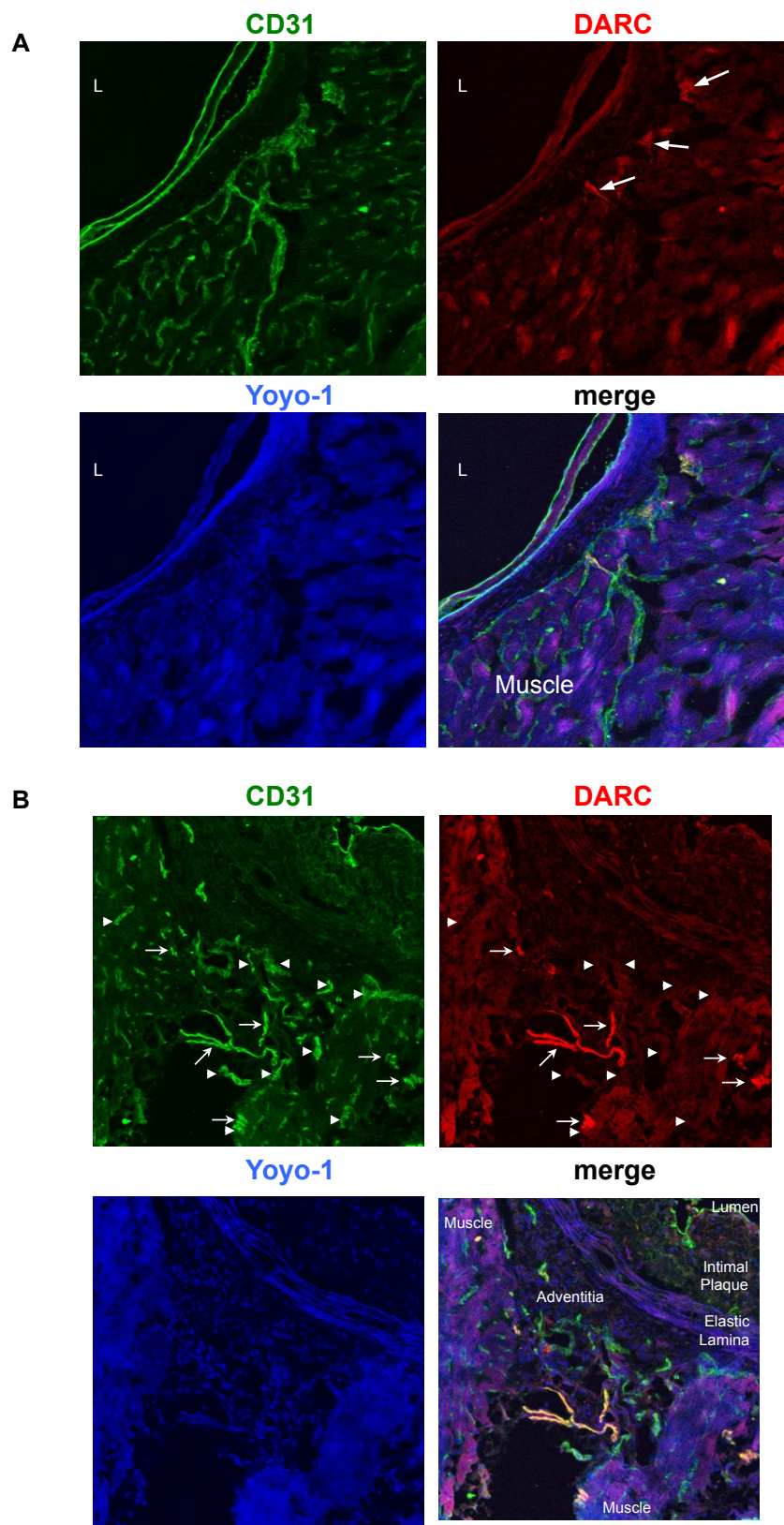

Supplement: Supplementary file 8 — DARC positive vessels in vasa vasorum of aorta of wildtype (WT) and Apoe –/– mice. Representative confocal micrographs of DARC expression on venules in the vasa vasorum of aorta of WT (A) or Apoe –/– mice (B). Tissues were stained with anti-CD31 (green), anti-DARC MAb (red), and Yoyo-1 (blue) was used to stain nuclei. Arrows indicate DARC+ vessels and arrowheads indicate DARC– vessels (WT n = 5 experiments, Apoe–/– n = 8 experiments). (PDF 426 kb) [file 12915_2017_381_MOESM6_ESM.pdf]

Supplemental Figure 6: DARC epitope sensitivity to enzymatic digestion

A

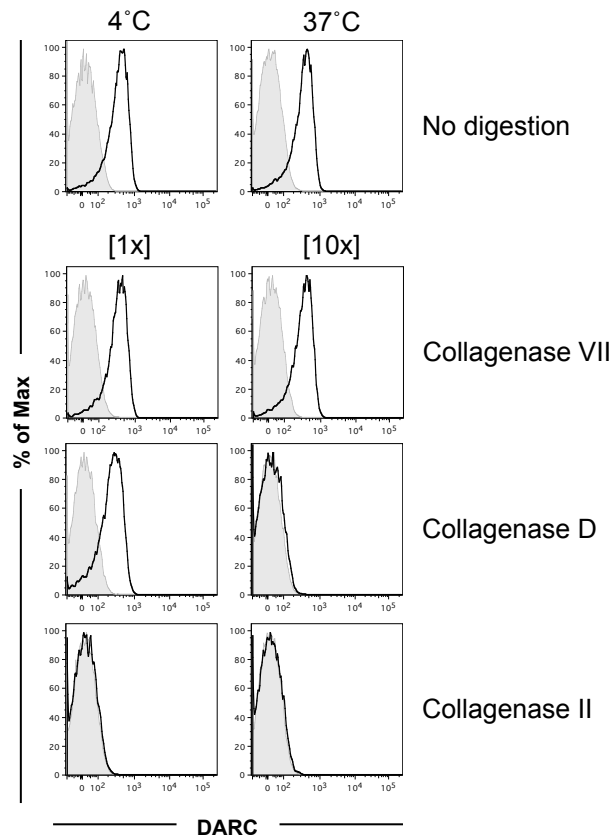

B

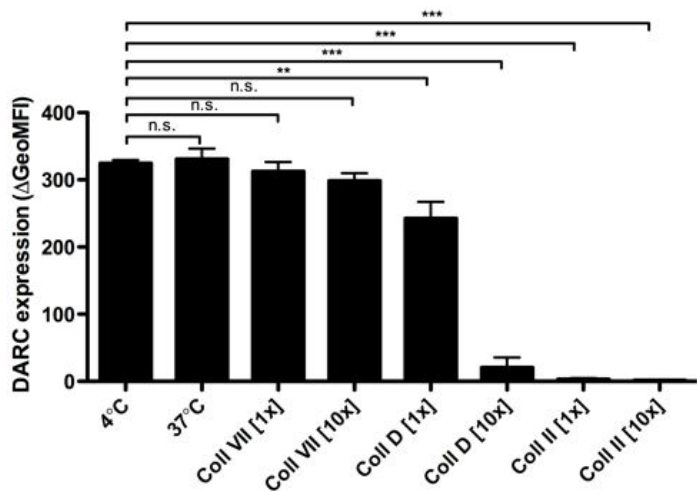

Supplement: Supplementary file 9 — DARC epitope sensitivity to enzymatic digestion. (A) Representative flow cytometry histograms of DARC expression on RBCs. RBCs from blood were digested at 37 °C with different concentration of enzymes as indicated: Collagenase VII (1×) = 86 μg/mL, Collagenase D (1×) = 2.5 mg/mL and Collagenase II (1×) = 1.5 mg/mL. RBCs at 4 °C and 37 °C without digestion are used as positive controls for DARC expression. Flow cytometry was performed to detect DARC expression (n = 5 experiments). (B) Quantification of DARC expression on RBCs, results are shown as delta Geometric Mean Intensity of Fluorescence (n = 3 mice/group). Error bars show mean ± SEM. ns P > 0.05, **P ≤ 0.01, ***P ≤ 0.001. Supporting data values are included in Additional file 2. (PDF 180 kb) [file 12915_2017_381_MOESM7_ESM.pdf]

## Supplemental Figure 8: DARC expression on lymphatic endothelial cells

**A** LECs gated on  $CD45^-CD31^+gp38^+$

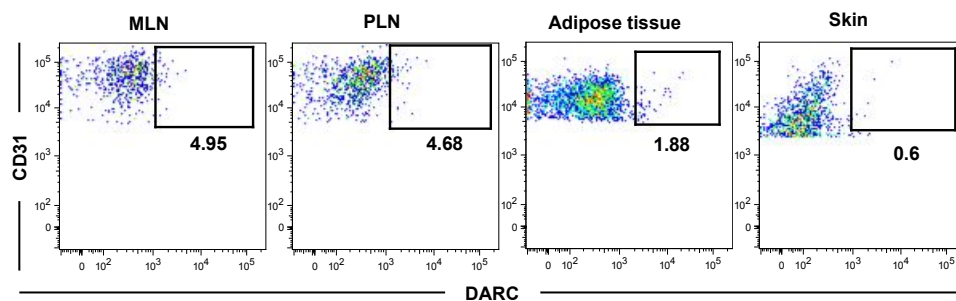

**B**

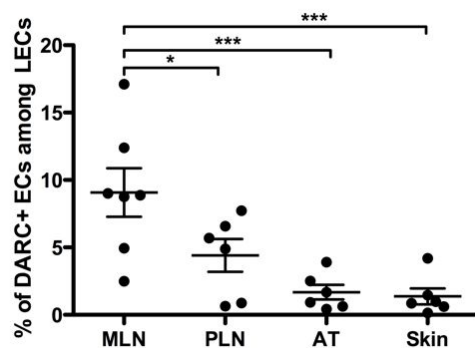

**C**

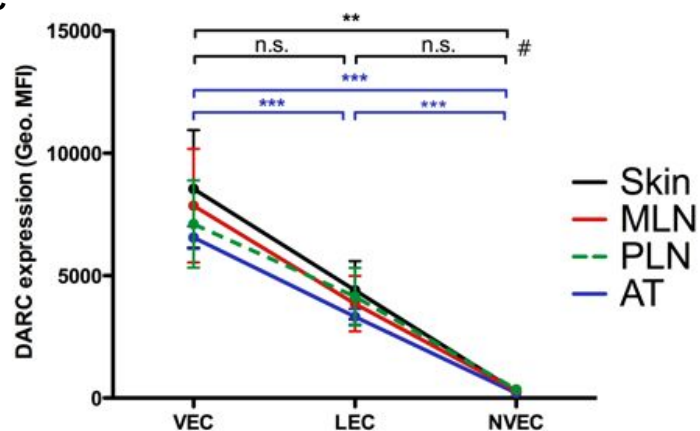

Supplement: Supplementary file 11 — DARC expression on lymphatic endothelial cells. (A) Flow cytometry analysis of DARC expression on lymphatic endothelial cells (LECs) in mesenteric lymph node (MLN), peripheral lymph node (PLN), adipose tissue, and skin. Live LECs are defined as followed: CD45 negative, CD31 and gp38 positive. DARC expression is shown as frequency of LEC subset. (B) Bar graph showing the frequency of DARC+ LECs among total LEC population in MLN (n = 7), PLN (n = 6), adipose tissue (AT) (n = 6), and skin (n = 6). Data were excluded from the analysis if number of events in LEC gate was less than 500. Error bars show mean ± SEM. *P ≤ 0.05, ***P ≤ 0.001. (C) DARC expression shown as geometric mean of intensity of fluorescence (Geo.MFI) on venules (V-EC), lymphatic EC (LEC), and non-venules (NV-EC). Error bars show mean ± SEM. ns P > 0.05, **P ≤ 0.01, ***P ≤ 0.001. “#” Identical statistic results for skin, MLN, and PLN. Statistic results for AT are indicated in blue. (PDF 186 kb) [file 12915_2017_381_MOESM9_ESM.pdf]
