# Supplementary material for: Differential DARC/ACKR1 expression distinguishes venular from non-venular endothelial cells in murine tissues
Source: BMC Biol. 2017 May 19;15:45. doi: 10.1186/s12915-017-0381-7 (PMC5438556; doi:10.1186/s12915-017-0381-7)

Supplemental Figure 3: Quantification of DARC expression on blood microvasculature

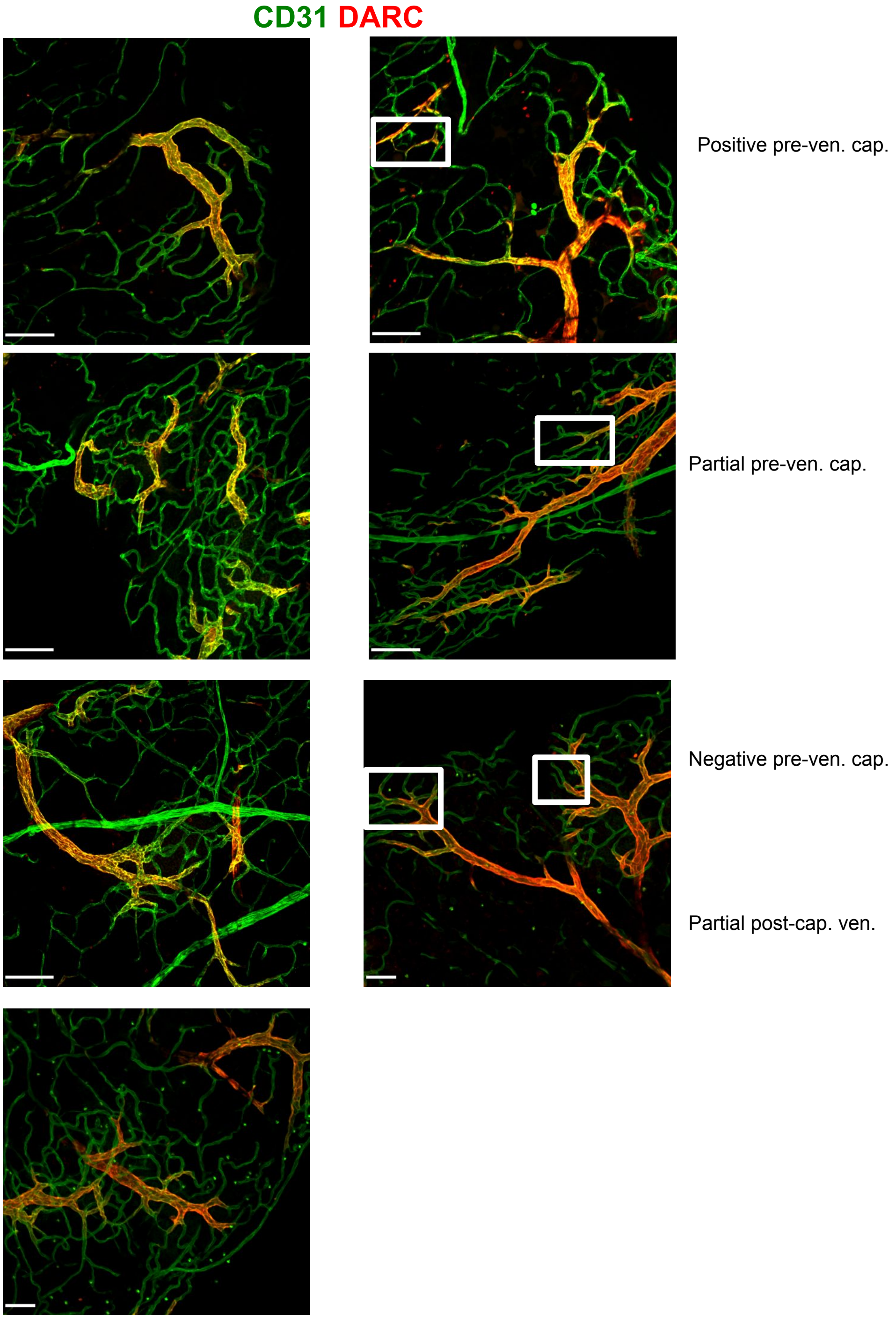

Supplement: Supplementary file 6 — Quantification of DARC expression on blood microvasculature. To determine DARC expression on arterioles, capillaries, pre-venular capillaries (PVC), post-capillary venules (PCV), and collecting venules, we analyzed DARC expression in a microvascular network stained with anti-CD31 (green) and anti-DARC (red). White squares indicate the regions selected to illustrate positive, partial, or negative pre-venular capillaries (PVC) for DARC expression as well as partial DARC expression on post-capillary venules (PCV) in Fig. 2; 20× objective, scale bars = 200 μm. (PDF 391 kb) [file 12915_2017_381_MOESM4_ESM.pdf]
