# Supplementary material for: Differential DARC/ACKR1 expression distinguishes venular from non-venular endothelial cells in murine tissues
Source: BMC Biol. 2017 May 19;15:45. doi: 10.1186/s12915-017-0381-7 (PMC5438556; doi:10.1186/s12915-017-0381-7)

Supplemental Figure 7: DARC expression in whole mount adipose tissue

CD31 DARC

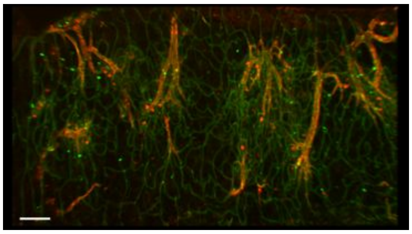

Surface area for CD31

3161698.05 μm^2

Surface area for DARC

464211.96 μm^2

Merge of surface areas

14.68% overlap

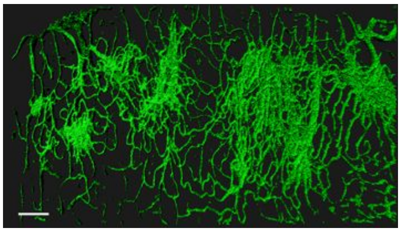

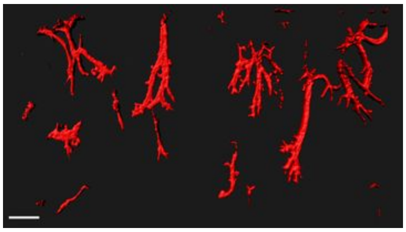

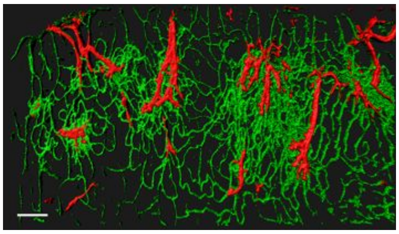

CD31 DARC

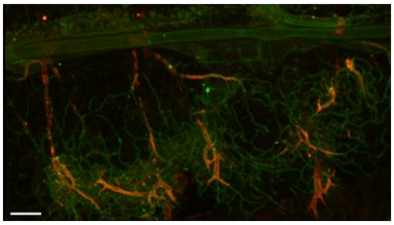

Surface area for CD31

2187833.23 μm^2

Surface area for DARC

301388.47 μm^2

Merge of surface areas

13.78% overlap

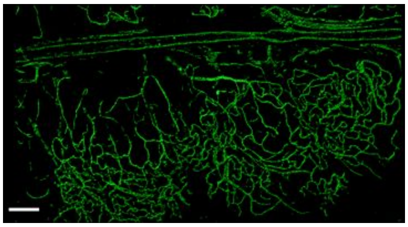

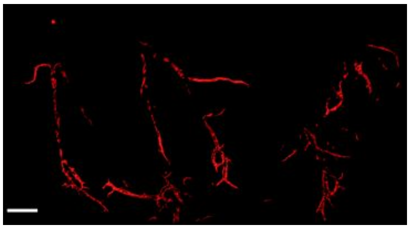

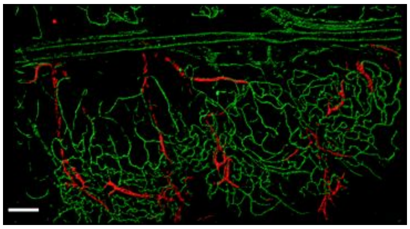

Supplement: Supplementary file 10 — DARC expression in whole mount adipose tissue. Confocal micrographs of whole mount staining of adipose tissue (omentum) were analyzed for CD31 (green) and DARC (red) expression. The fluorescence intensity images (upper panels) and the corresponding 3D rendering images (lower panels) are shown. Surface area for CD31 and DARC channels were calculated using Imaris software and shown as μm2. 10× objective, scale bars = 100 μm. Supporting data values are included in Additional file 2. (PDF 261 kb) [file 12915_2017_381_MOESM8_ESM.pdf]
